# Orphan Nuclear Receptor Errγ Induces C-Reactive Protein Gene Expression through Induction of ER-Bound Bzip Transmembrane Transcription Factor CREBH

**DOI:** 10.1371/journal.pone.0086342

**Published:** 2014-01-22

**Authors:** Jagannath Misra, Dipanjan Chanda, Don-Kyu Kim, Seung-Rye Cho, Seung-Hoi Koo, Chul-Ho Lee, Sung Hoon Back, Hueng-Sik Choi

**Affiliations:** 1 National Creative Research Initiatives Center for Nuclear Receptor Signals, Hormone Research Center, School of Biological Sciences and Technology, Chonnam National University, Gwangju, Republic of Korea; 2 Division of Life Sciences, College of Life Sciences and Biotechnology, Korea University, Seoul, Republic of Korea; 3 Korea Research Institute of Bioscience and Biotechnology, Daejeon, Republic of Korea; 4 School of Biological Sciences, University of Ulsan, Ulsan, South Korea; 5 Research Institute of Medical Sciences, Department of Biomedical Sciences, Chonnam National University Medical School, Gwangju, Republic of Korea; Institut de Génomique Fonctionnelle de Lyon, France

## Abstract

The orphan nuclear receptor estrogen-related receptor-γ (ERRγ) is a constitutively active transcription factor regulating genes involved in several important cellular processes, including hepatic glucose metabolism, alcohol metabolism, and the endoplasmic reticulum (ER) stress response. cAMP responsive element-binding protein H (CREBH) is an ER-bound bZIP family transcription factor that is activated upon ER stress and regulates genes encoding acute-phase proteins whose expression is increased in response to inflammation. Here, we report that ERRγ directly regulates CREBH gene expression in response to ER stress. ERRγ bound to the ERRγ response element (ERRE) in the CREBH promoter. Overexpression of ERRγ by adenovirus significantly increased expression of CREBH as well as C-reactive protein (CRP), whereas either knockdown of ERRγ or inhibition of ERRγ by ERRγ specific inverse agonist, GSK5182, substantially inhibited ER stress-mediated induction of CREBH and CRP. The transcriptional coactivator PGC1α was required for ERRγ mediated induction of the CREBH gene as demonstrated by the chromatin immunoprecipitation (ChIP) assay showing binding of both ERRγ and PGC1α on the CREBH promoter. The ChIP assay also revealed that histone H3 and H4 acetylation occurred at the ERRγ and PGC1α binding site. Moreover, chronic alcoholic hepatosteatosis, as well as the diabetic obese condition significantly increased CRP gene expression, and this increase was significantly attenuated by GSK5182 treatment. We suggest that orphan nuclear receptor ERRγ directly regulates the ER-bound transcription factor CREBH in response to ER stress and other metabolic conditions.

## Introduction

Estrogen-related receptors (ERRs) are members of the NR3B subfamily of nuclear receptors which include ERRα, ERRβ, and ERRγ. These orphan nuclear receptors regulate transcription via ERREs but do not bind endogenous estrogen [1]. The ERRs are named due to the conservation in the structure of their DNA-binding domains/DBDs with the highly homologous Estrogen Receptor [2]. Crystallographic studies indicate that the ERRs along with ERRγ are constitutively active without a natural ligand, while several synthetic ligands either stimulate or repress the activity of ERRγ by promoting or disrupting ERR–coactivator interactions [3]. Among them GSK5182, a 4-hydroxy tamoxifen analogue, is a selective inverse agonist of ERRγ and directly binds to the ligand binding domain and inhibits transactivation by ERRγ [4]–[8]. ERRγ is primarily expressed in heart, brain, kidney, pancreas and liver tissues [3]. We previously reported that hepatic ERRγ regulates hepatic gluconeogenesis by directly binding to the Phosphoenolpyruvate carboxykinase (PEPCK) and Glucose 6-phosphatase (G6Pase) promoters along with coactivator PGC-1α [5]. Previous results from our laboratory also demonstrated that ERRγ directly binds to the LIPIN1 promoter along with coactivator PGC-1α to regulate LIPIN1 gene expression, and inhibits hepatic insulin signaling [6]. ERRγ also controls hepatic CB1 receptor-mediated CYP2E1 expression at the transcriptional level and thus contributes to the oxidative liver injury by alcohol [7]. Finally, hypoxia induces PDK4 gene expression through induction of ERRγ [8]. The transcriptional activity of the ERR family is dependent on interactions with coactivators, in particular PGC-1α and PGC-1β [9]. ERRα and ERRγ regulate mitochondrial programs involved in oxidative phosphorylation and a nuclear-encoded mitochondrial genetic network that coordinates the postnatal metabolic transition in the heart [9]. Though all these reports clearly suggest a key role of ERRγ in different cellular processes, its role in ER stress is yet to be determined.

ER stress is a state associated with perturbation of ER homeostasis and accumulation of unfolded or misfolded proteins in the ER [10]. CREBH, an ER-stress-activated liver enriched transcription factor, has been previously reported to transcriptionally activate acute phase response genes in the liver in response to lipopolysaccharide (LPS) and pro-inflammatory cytokines interleukin-6 (IL-6) and tumor necrosis factor α (TNFα) [11]. Recently, CREBH has been demonstrated to play a critical role in ER-stress-mediated regulation of iron metabolism via induction of hepcidin (Hamp) gene expression, in triglyceride metabolism and hepatic lipogenesis, and in the mediation of the hormonal regulation of hepatic gluconeogenesis under fasting or insulin-resistant conditions [12]–[15], thereby underlining the importance of CREBH in various hepatic metabolic pathways. Recent studies from our group have demonstrated that activation of Cb1r leads to phosphorylation of the c-Jun N-terminal Kinase (JNK) signaling pathway which in turn activates CREBH. This Cb1r-JNK-CREBH pathway was further demonstrated to regulate hepatic gluconeogenesis by regulating key gluconeogenic genes (PEPCK, and G6Pase) and lipid metabolism by regulating Lipin1 [16]–[17]. Our group has also reported that hepatic cannabinoid receptor type 1 mediates alcohol-induced regulation of bile acid enzyme genes (CYP7A1, and CYP27A1) expression via CREBH [18].

PGC-1α, a member of a small family of coactivators, was identified using yeast two hybrid assays for PPARγ-interacting proteins [19] and is implicated in mitochondrial metabolism, thermogenesis, mitochondrial biogenesis, adipocyte differentiation, gluconeogenesis and glucose uptake [20]–[21]. PGC-1α targets promoters by interacting directly with numerous DNA binding transcription factors and then coordinating several biochemical events, including recruitment of chromatin modifying enzymes such as p300/CBP and SRC-1, interaction with the basal transcription machinery and linking of transcription to RNA splicing [22].

C-reactive protein (CRP) in comparison to other inflammation markers is a relatively stable, robust and an exquisitely sensitive serum protein. In some studies, CRP level in plasma predicts cardiovascular events even better than low-density lipoprotein cholesterol level [23]. Multiple epidemiological and mechanistic studies have shown that CRP is not just a marker but rather an active mediator for endothelial dysfunction, arterial thrombosis and atherogenesis [24]–[25]. CRP, as an inflammatory cytokine, is primarily synthesized in liver and regulated in response to interleukin-6 (IL-6) and interleukin-1β (IL-1β) [26]–[28]. Hepatocytes are believed to be the major contributor of circulating CRP in plasma. Therefore, hepatic CRP is able to cause liver per se damage and inflammation, and plays a key role in the development of atherosclerosis when entering into the blood circulation. In hepatoma cell lines, the endogenous CRP gene is either dysregulated or weakly active [29]. However, plasma levels of CRP may rise rapidly and markedly (>1000 fold) after an acute inflammation in human [30].

Here, we examined the mechanism of how nuclear receptor, ERRγ regulates ER bound bZIP transcription factor, CREBH under the conditions of ER stress. Upon ER stress, expression of both transcription factors increases significantly. ERRγ directly binds to the CREBH promoter. Of interest, PGC1α acts as a coactivator for ERRγ. We observed a significant increase in histone H3 and H4 acetylation in CREBH promoters upon induction of ER stress by tunicamycin (Tm) treatment. Knockdown of ERRγ significantly decreases the expression of CREBH and its target gene CRP. Together, we present a novel mechanistic pathway that would encourage further study to elucidate the relation between nuclear receptors and ER stress.

## Materials and Methods

### Ethics Statement

All procedures were approved by the Institutional Animal Care and Use Committee (IACUC) in Korea Research Institute of Bioscience and Biotechnology (KRIBB).

### Animal Experiments

Male 7–12-week-old C57BL/6J mice or *db*/*db* mice (Charles River Laboratories) were acclimatized to a 12 hr light–dark cycle at 22±2°C with free access to food and water in a specific pathogen-free facility. Tunicamycin (Tm) (1 mg/kg, i.p. in 1% DMSO/DW) was administered by intraperitoneal injection into C57BL/6J mice (n = 5 per group) or Ad-GFP or Ad-ERRγ were injected via tail-vein into male C57BL/6J mice (n = 5 per group). Where indicated, GSK5182 was administered first (40 mg/kg, p.o. in 30% PEG400/DW) by intraperitoneal injection and after 30 minutes Tm (1 mg/kg, i.p. in 1% DMSO/DW) was administered by intraperitoneal injection into C57BL/6J mice (n = 5 per group). For the chronic alcoholic hepatosteatosis model, four groups of five mice each were treated with alcohol-containing Lieber-DeCarli formulation based liquid (Dyets, Bethlehem, Pennsylvania, USA) diet (27.5% of total calories) for 4 weeks. For alcohol+GSK5182 treatment, during the 4 weeks of feeding with alcohol (27.5% of total calories) liquid diet, GSK5182 (40 mg/kg, oral, once-daily) was injected for the last 2 weeks into mice. For diabetic mouse study, GSK5182 (40 mg/kg/day as a final dose) and corn oil emulsion were sonicated again immediately before injection of *db*/*db* mice. After 14 hr. of fasting, intraperitoneal injections were performed for 5 days. All experiments were conducted as previously described [7].

### Chemicals and Antibodies

Tm was obtained from Sigma-Aldrich and, GSK5182 was synthesized as described previously [6]. Antibodies used in this work were as follows: anti-ERRγ (Perseus Proteomics), anti-tubulin (Ab_FRONTIER_), anti-PGC1α (Santa Cruz), anti-acetyl-histone H3 (Cell Signaling), anti-acetyl-histone H4 (Cell Signaling), and anti-CRP (Abcam). Anti-CREBH antibody was described previously [15]. The primary antibodies were used at a dilution ranging from 1∶200 to 1∶1000 for western blot analyses, and at a dilution of 1∶200 for immunoprecipitation.

### Plasmids and Adenovirus

All mouse CREBH promoters were cloned from mouse genomic DNA and inserted into pGL3-Basic vector using Xho1/Hind3 restriction sites. Mutant CREBH promoter was made using wild-type CREBH promoter (−0.45 kb) as template by Quick Change Lightning Site-Directed Mutagenesis kit from Agilent Technologies using primres, antisense: 5'-cgggtctgtgtgaaatctcccctacttacagctactgttt-3', and sense: 5'-aaacagtagctgtaagtaggggagatttcacac-agacccg-3'. Human CREBH promoter was cloned from human genomic DNA and inserted into pGL3-Basic vector using Xho1/Hind3 restriction sites. CRP promoter containing reporter [11], expression vector for FLAG-ERRγ [7] and, PGC1α [6] were described previously. All plasmids were confirmed via DNA sequence analyses. For ectopic expression of the genes, adenoviral delivery was used. Adenoviruses (Ad) encoding GFP only (Ad–GFP), Ad–ERRγ, Ad–ERRα, Ad–shERRγ, Ad-PGC1α, Ad-CREBHi and, Ad-shPGC1α were described elsewhere [6]–[7], [15], [31].

### Cell Culture, Transient Transfection and Luciferase Assay

AML12 (mouse hepatoma cell line), HepG2 (human hepatoma cell line) and, 293T (human embryonic kidney cell line) cells were obtained from the American Type Culture Collection. Maintenance of cell lines and transient transfection assays were performed using Lipofectamine 2000 transfection reagent (Invitrogen) according the manufacturer’s instructions as described elsewhere [32]. Briefly, cells were transfected with indicated reporter plasmids together with expression vectors encoding various transcription factors or treated with various chemicals. Total cDNA used for each transfection was adjusted to 1 µg/well by adding appropriate amount of empty vector and pCMV–β-gal plasmid was used as an internal control. The luciferase activity was normalized to β-galactosidase activity and expressed as relative luciferase units (RLU).

### RNA Interference

Knockdown of ERRγ and CREBH was performed using the pSuper vector system [33]–[34]. AML12 cells were transfected with siRNA constructs using Lipofectamine 2000 (Invitrogen) according to the manufacturer’s instructions. siRNA treated cells were analyzed reverse transcription PCR (RT–PCR) to measure the extent of knockdown.

### Reverse Transcriptase PCR and Quantitative Real-time PCR Analyses

Total RNA was isolated using the TRIzol reagent (Invitrogen) according to the manufacturer’s instructions. The mRNAs of ERRγ, CREBH, CRP, and PGC1α were analyzed by reverse transcription PCR (RT–PCR) or quantitative real-time RT-PCR (qPCR) as indicated. DNA samples from total RNA reverse transcription assays served as the templates for qPCR, which were performed with TOPreal SYBR Green PCR Kit (Enzynomics) and the Step One Plus real-time PCR system (Applied Bioscience) in triplicate. mRNA expression levels were normalized to those of β-actin (ACTB). Primers Sequence (5′–3′) used for PCR are as follows. mCREBH FP: GTGTCACACCAGGGAGCAAG, mCREBH RP: CAGTGAGGTTGAAGCGGGAG, mCRP FP: GGCCAGATGCAAGCATCATC, mCRP RP: CTGGAGATAGCACAAAGTCCCAC, mCREBH promoter FP (–195/−87): GTGACGCTAGACAG, mCREBH promoter RP (–195/−87): GTGCTTTTTCCAGG, mCREBH promoter FP (–766/−642): GGGTTACAGGAGTAA, mCREBH promoter RP (–766/−642): ACAAACTCTCTGCC.

### Chromatin Immunoprecipitation Assay

Formaldehyde cross-linking of cells, and chromatin immunoprecipitations (ChIPs) analyses were performed as described elsewhere [5].

### Statistical Analyses

Data are expressed as means±SEM. Statistical analyses was performed using the two-tailed Student t test. Differences were considered statistically significant at p<0.05.

## Results

### ERRγ Induces CREBH Gene Expression

**Figure 1:**
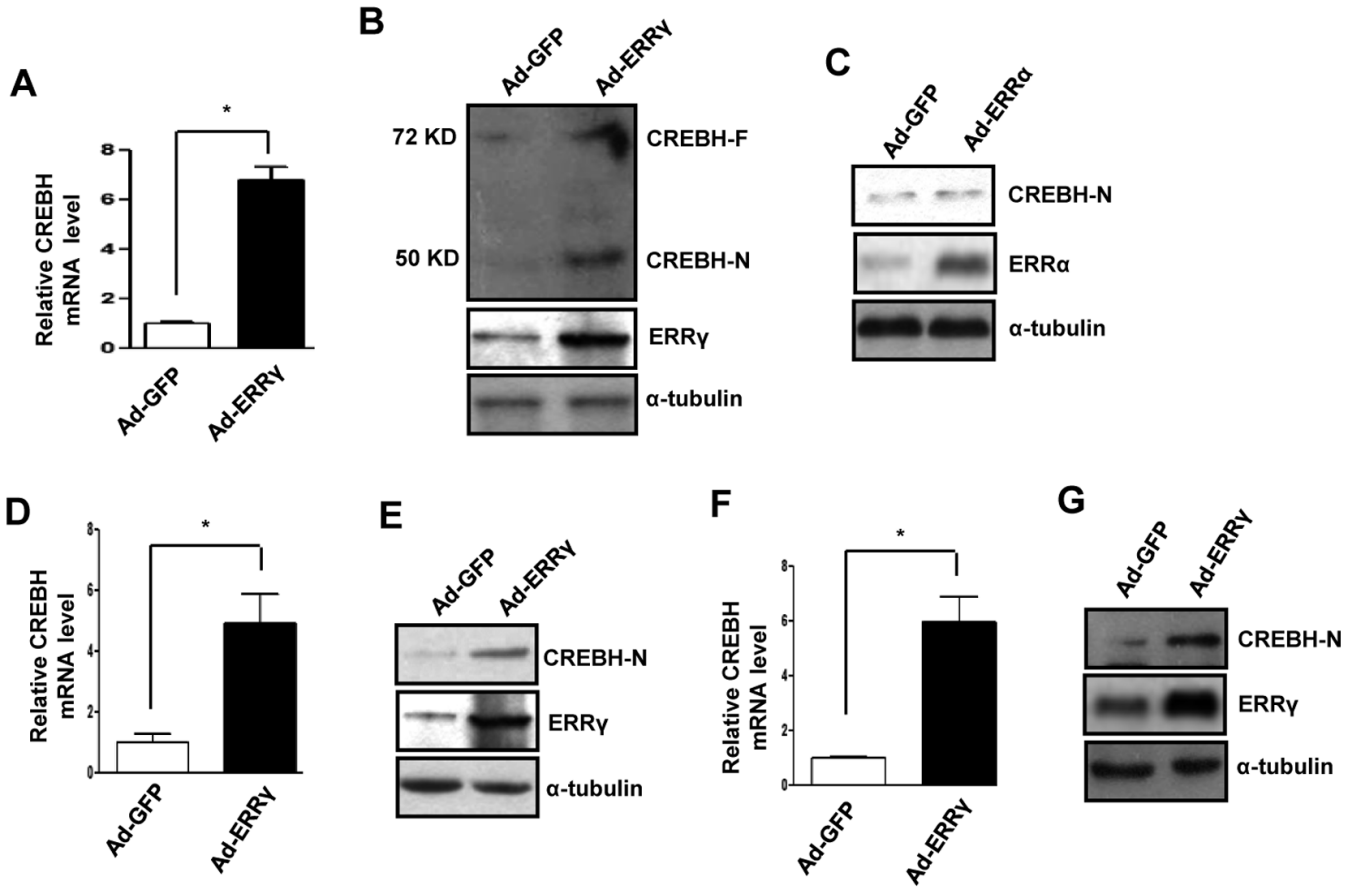
ERRγ induces CREBH gene. (A,B), AML12 cells were infected with Ad-GFP or Ad-ERRγ for 24 hr. (A),Total RNA was isolated for qRT-PCR analyses to quantify CREBH mRNA levels using CREBH primers. (B), Western blot analyses shows CREBH full length (CREBH-F), and CREBH active form (CREBH-N) expression. (C), AML12 cells were infected with Ad-GFP or Ad-ERRα for 24 hr. Western blot analyses shows CREBH-N expression. (D,E), Ad-GFP or Ad-ERRγ were injected via tail vein into male C57BL/6J mice (n = 5 per group). Following completion of the experiments, mice were sacrificed, and liver tissues were obtained for (D), qRT-PCR analyses to quantify CREBH mRNA levels, and (E), western blot analyses of CREBH-N expression. (F,G), HepG2 cells were infected with Ad-GFP or Ad-ERRγ for 24 hr. (F),Total RNA was isolated for qRT-PCR analyses to quantify CREBH mRNA levels using CREBH primers. (G), Western blot analyses shows CREBH-N expression. Data are representative of three independently performed experiments and shown as mean±SD; *, P<0.05 using Student’s t-test.

Previous reports suggest various nuclear receptors and transcription factors cross talk to regulate the mammalian ER stress response [35]–[36]. In addition, the nuclear receptors GR, PPARα, and HNF4α regulate transcription of the ER bound transcription factor CREBH [15], [36]–[37]. We demonstrated previously that ERRγ regulates ATF6α in response to ER stress [38]. To investigate whether ERRγ regulates CREBH, which belongs to the same CREB3 family as ATF6 and is structurally related to it, ERRγ was overexpressed by Ad-ERRγ in AML12 cells. Interestingly, CREBH mRNA level, as determined by qPCR, increased more than six-fold after ERRγ overexpression (Fig. 1A). CREBH protein level, as determined by Western blot, also increased under the same experimental conditions (Fig. 1B). Next, to check whether CREBH gene induction was ERRγ specific, ERRα was overexpressed in AML12 cells by adenovirus. No significant change in CREBH protein level was observed after ERRα overexpression, suggesting that CREBH gene induction is ERRγ specific (Fig. 1C). To verify these *in vitro* results, mice were infected with Ad-ERRγ by tail vein injection. Consistent with the *in vitro* results, an almost fivefold increase in CREBH mRNA level was observed. A significant increase in CREBH-N (active form) protein level was also observed *in vivo* (Fig. 1D and 1E). As observed in the mouse cell lines, we noticed a similar effect of ERRγ overexpression in HepG2 cells. ERRγ overexpression significantly increased CREBH mRNA, and protein levels (Fig. 1F and G), indicating that ERRγ-mediated induction of the CREBH gene is conserved in humans and in mouse hepatoma cell lines. Collectively these results demonstrate that ERRγ positively regulates CREBH gene expression.

### ER Stress Induces CREBH Gene Expression via ERRγ

**Figure 2:**
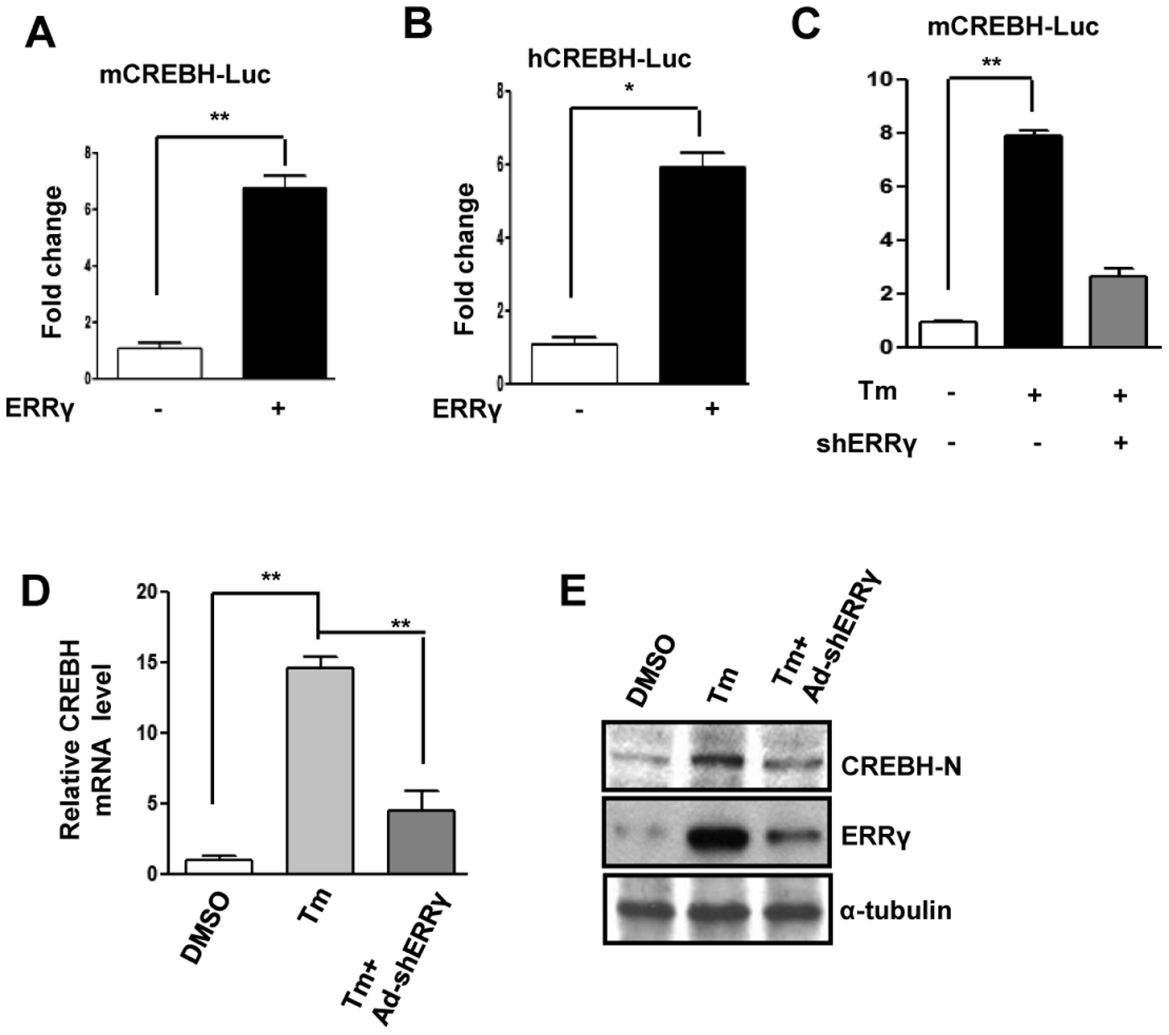
ER stress induces CREBH gene expression via ERRγ. (A), Activation of the mouse CREBH promoter by ERRγ. Transient transfection was performed in 293T cells with the indicated plasmid DNAs. (B), Activation of the human CREBH promoter by ERRγ. Transient transfection was performed in 293T cells with the indicated plasmid DNAs. (C), Effect of ERRγ knock down on the mouse CREBH promoter activation by ER stress. Transient transfection was performed in 293T cells with the indicated plasmid DNAs. (D, E), AML12 cells were treated with DMSO or Tm (5µg/mL) alone for 12 hr. or first infected with Ad-shERRγ, and at 48 hr. after infection treated with Tm (5µg/mL) for 12 hr. (D), Total RNA was isolated for qRT-PCR analyses to quantify CREBH mRNA levels using CREBH primers, and (E), Western blot analyses shows ERRγ and CREBH-N expression. Data are representative of three independently performed experiments and shown as mean±SD; *, P<0.05, and **, P<0.005 using Student’s t-test.

Next, we ascertained the molecular mechanism of ERRγ-mediated CREBH gene induction. A transient transfection assay was performed with the ERRγ expression vector and the mouse and human CREBH promoter- driven luciferase reporter gene (Fig. 2A, and B). ERRγ significantly enhanced both mouse and human CREBH promoter activity. It was reported previously that tunicamycin (Tm) induces CREBH and ERRγ gene expression [11], [38]. To investigate whether Tm mediated activation of the CREBH promoter depends on ERRγ, a transient transfection assay was performed with the mouse CREBH promoter-driven luciferase reporter gene. Tm treatment significantly increased CREBH promoter activity but activation was severely compromised when endogenous ERRγ was knocked down (Fig. 2C). Next, AML12 cells were treated with Tm or infected with Ad-shERRγ to knockdown endogenous ERRγ to observe the effect of ERRγ knockdown on CREBH gene expression. As expected, knockdown of endogenous ERRγ significantly suppressed the Tm-mediated increase in CREBH mRNA level (Fig. 2D). Next, we performed Western blot analyses to check the CREBH-N protein level under the same experimental conditions. In agreement with the mRNA level, CREBH-N protein level also decreased significantly in response to Ad-shERRγ infection (Fig. 2E) in AML12 cells. Taken together, these results demonstrate that ERRγ mediates the induction of CREBH gene expression by ER stress.

### ERRγ Activates CREBH Gene Promoter via an ERRE

**Figure 3:**
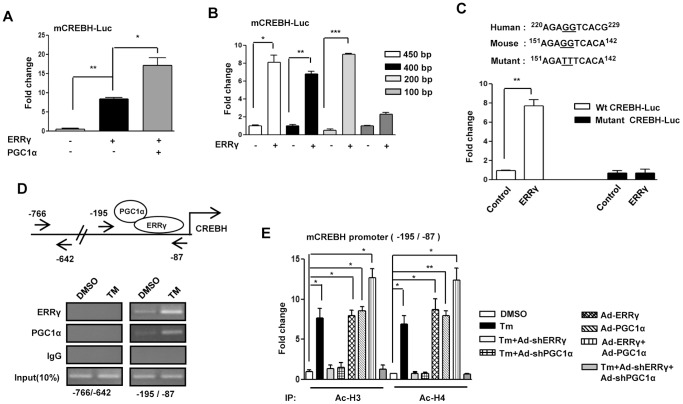
ERRγ regulates activation of CREBH gene promoter. (A), PGC1α-dependent activation of the mouse CREBH promoter by ERRγ. Transient transfection was performed in 293T cells with the indicated plasmid DNAs. (B), Deletion constructs of the CREBH promoter demonstrate the ERRγ binding site in 293T cells. Transient transfection was performed in 293T cells with the indicated plasmid DNAs. (C), ERRE-dependent activation of the CREBH promoter in 293T cells. 293T cells were transfected with the wild-type or ERRE-mutant CREBH promoter along with ERRγ plasmid DNAs. (D), ChIP assay shows the binding of ERRγ and PGC1α to the endogenous CREBH promoter by semiquantitative PCR. AML12 cells were treated with DMSO or Tm (5µg/mL) for 12 hr. After completion of the treatment, chromatin fragments were prepared and immunoprecipitated with ERRγ, PGC1α, or IgG control antibodies. DNA fragments covering –766 to –642 and –195 to –87 elements on the CREBH promoter were PCR amplified. 10% of the soluble chromatin was used as input. (E), ChIP assay for detection of histone acetylation at the ERRγ/PGC1α binding site under the indicated conditions in AML12 cells. Chromatin fragments were prepared and immunoprecipitated with Acetyl-Histone 3 and Acetyl-Histone 4 antibodies. DNA fragments covering –195 to –87 element on the CREBH promoter were qPCR-amplified as described in the ‘Materials and Methods’ section. Data are representative of three independently performed experiments and shown as mean±SD; *P<0.05, **P<0.005, and ***, p<0.0005 using Student’s t-test.

PGC1α acts as an ERRγ coactivator [6]. To test whether PGC1α and ERRγ together play any role in the induction of CREBH, transient transfection assays were performed with the mouse CREBH promoter-driven luciferase reporter gene and ERRγ and PGC1α expression vectors. ERRγ significantly increased CREBH promoter activity, and this activity was further augmented in the presence of PGC1α (Fig. 3A). A series of deletion constructs was analyzed to identify the DNA sequence conferring the ERRγ-mediated ER stress effect on the CREBH promoter. Deletion of the CREBH promoter sequence from 0.2 kb to 0.1 kb drastically decreased the promoter activity conferred by ERRγ, suggesting that the region from 0.2 kb to 0.1 kb confers activation of the CREBH promoter (Fig. 3B). It was reported previously that ERRγ binds to a consensus sequence AGGTCA [38]. We aligned this sequence with the CREBH promoter region from 0.2 kb to 0.1 kb and found a perfect match to the consensus sequence, AGGTCA, spanning the 0.2 kb to 0.1 kb region. Transient transfection assays were performed using wild-type and AGGTCA mutant CREBH promoters with the ERRγ expression vector to test whether AGGTCA is critical for ERRγ binding. This mutant reporter did not show any significant response to ERRγ cotransfection (Fig. 3C). Next, the ChIP assay was performed to monitor the effect of Tm on ERRγ and PGC1α recruitment to the endogenous CREBH gene promoter. Under basal conditions, both ERRγ and PGC1α occupied the CREBH promoter. However, Tm treatment significantly augmented ERRγ and PGC1α occupancy on the CREBH promoter (Fig. 3D). The ChIP assay results provide critical *in vivo* evidence that the ER-stress-ERRγ-PGC1α signaling pathway increases CREBH gene transcription. Gene activation is often associated with increased histone acetylation [39], and the ChIP assay was performed to determine whether Tm treatment results in increased template-associated histone (H3 and/or H4) acetylation of the CREBH gene promoter in AML12 cells (Fig. 3E). Tm treatment and adenoviral overexpression of ERRγ and/or PGC1α increased acetylation of H3 (Ac–H3) and H4 (Ac–H4) on the ERRγ-responsive region of the CREBH promoter, whereas knockdown of endogenous ERRγ or PGC1α significantly reduced histone (H3 and/or H4) acetylation. We observed a synergistic effect of ERRγ and PGC1α overexpression on acetylation of H3 (Ac–H3) and H4 (Ac–H4) (Fig. 3E) which was in accordance with our previous results (Fig. 3A) where, synergistic effect on CREBH promoter activation was clearly observed by cotransfection of ERRγ and PGC1α with CREBH promoter-driven luciferase reporter gene. Overall, these results demonstrate that ER stress augments the binding of ERRγ and PGC1α to the CREBH promoter and increases template-associated histone (H3 and H4) acetylation to facilitate CREBH gene transcription.

### Effect of ERRγ Knockdown on CREBH-mediated Regulation of CRP Gene Expression

**Figure 4:**
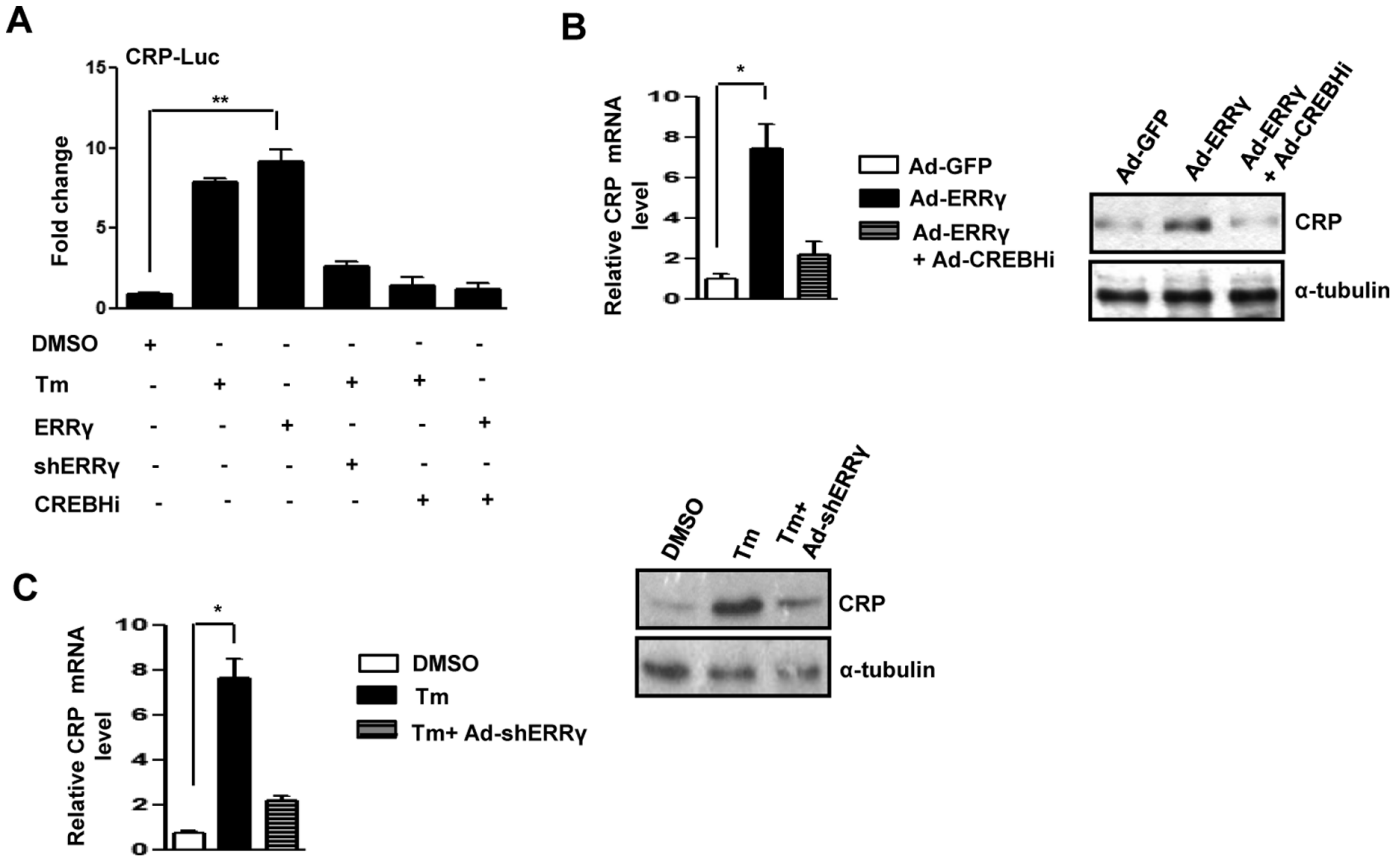
ERRγ regulates CREBH target gene. (A), ERRγ dependent activation of the CRP promoter by ER stress. Transient transfection was performed in 293T cells with the indicated plasmid DNAs. (B), AML12 cells were infected with Ad-GFP or Ad-ERRγ for 24 hr. or first infected with Ad-CREBHi, and at 48 hr. after, infected with Ad-ERRγ for 24 hr. (Left panel), total RNA was isolated for qRT-PCR analyses to quantify CRP mRNA levels using CRP primers, and (right panel), western blot analyses shows CRP expression. (C), AML12 cells were treated with DMSO or Tm (5µg/mL) alone for 12 hr. or first infected with Ad-shERRγ, and at 48 hr. after infection, treated with Tm (5µg/mL) for 12 hr. (Left panel), Total RNA was isolated for qRT-PCR analyses to quantify CRP mRNA levels using CRP primers, and (right panel), Western blot analyses shows CRP expression. Data are representative of three independently performed experiments and shown as mean±SD; *, p<0.05, and **, P<0.005 using Student’s t-test.

Next, we examined regulation of CREBH target genes. Our results (Figs. 1–3) demonstrate that ERRγ regulates CREBH gene expression in response to ER stress. CRP is a well-known marker of various human diseases and is regulated by CREBH in response to ER stress [11]. We examined the effect of ERRγ knockdown on Tm mediated CRP promoter activation by transient transfection assay in 293T cells to verify the effect of ERRγ on CREBH target gene (Fig. 4A). Both Tm treatment and ERRγ overexpression significantly increased CRP promoter activation. However, knockdown of endogenous ERRγ or CREBH significantly blunted the increase in CRP promoter activation by Tm. These results demonstrate that Tm-induced CRP promoter activation is mediated through ERRγ. Next, ERRγ was overexpressed in AML12 cells to confirm the effect of ERRγ on CRP gene expression (Fig. 4B). As expected, ERRγ overexpression significantly increased both CRP mRNA and protein levels, and this increase was significantly attenuated upon endogenous CREBH knockdown. This result indicates that ERRγ regulates CRP gene expression through CREBH. Endogenous ERRγ was knocked down in AML12 cells for further verification. In agreement with the previous results, we observed that the Tm-mediated increase in CRP mRNA and protein was substantially reduced after ERRγ knockdown (Fig. 4C). Taken together, we demonstrated that ERRγ induces CREBH target genes by regulating CREBH itself.

### Alcohol Feeding and Diabetic Obesity Induce Hepatic CREBH and CRP Gene Expression via Hepatic ERRγ

**Figure 5:**
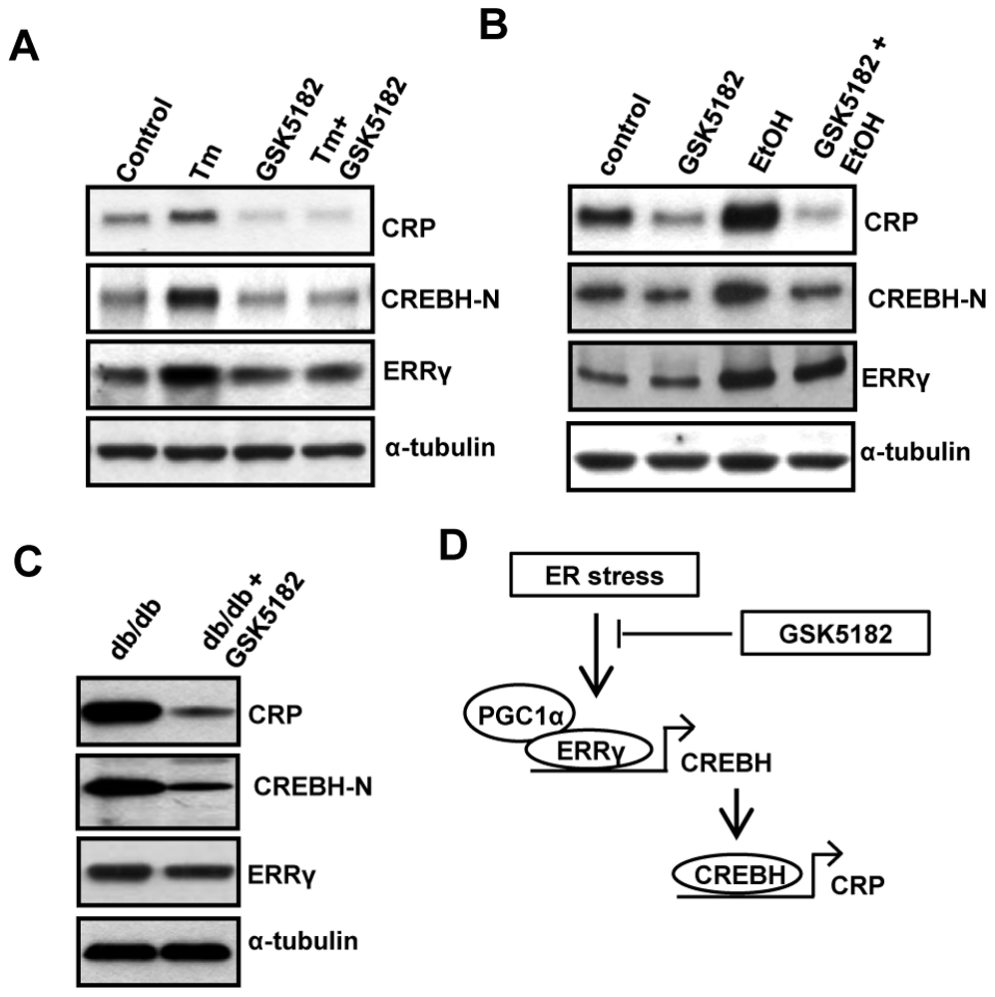
ERRγ inverse agonist inhibits CREBH and CRP *in vivo*. (A), GSK5182 was administered prior to (40 mg/kg, p.o. in 30% PEG400/DW) and at 30 minutes after Tm (1 mg/kg, i.p. in 1% DMSO/DW) was administered into C57BL/6J mice (n = 5 per group). Following completion of the experiments, mice were sacrificed, and liver tissues were obtained for western blot analyses of CRP, CREBH-N, and ERRγ. (B), GSK5182 was administered (40 mg/kg, p.o. in 30% PEG400/DW) into C57BL/6J mice (n = 5 per group) or EtOH was administered into C57BL/6J mice (n = 5 per group) or first GSK5182 was administered and then EtOH was administered (n = 5 per group). After completion of the treatment, mice were sacrificed. Western blot analyses shows CRP, CREBH-N and ERRγ expression in liver tissue. (C), GSK5182 was administered (40 mg/kg, p.o. in 30% PEG400/DW) into db/db mice (n = 5 per group) for 30 days. After completion of the treatment, mice were sacrificed. Western blot analyses shows CRP, CREBH-N and ERRγ expression in liver tissue. (D), Schematic representation of the proposed model in which ER stress mediated induction of CREBH is mediated through ERRγ.

Our results (Figs. 1–4) demonstrate that ER stress induces CREBH and CRP gene expression through ERRγ *in vitro*. GSK5182, an inverse agonist of ERRγ [4]–[8] that specifically binds to ERRγ and inhibits ERRγ transcriptional activity, was used to verify this effect *in vivo*. As expected from previous results, we noticed that GSK5182 treatment substantially reduced Tm-induced CREBH-N and CRP protein levels in mouse liver (Fig. 5A). Moreover, ERRγ protein levels were decreased upon Tm+GSK treatment as compared to Tm alone. This might be due to the mechanism of autoregulatory feed forward loop that regulates ERRγ gene expression [38]. Previous reports from our laboratory demonstrated that acute alcohol consumption induces hepatic ERRγ gene expression [8]. Mice were treated with EtOH to investigate whether ERRγ induces CREBH-N and CRP gene expression. The EtOH treatment significantly increased CREBH-N and CRP gene expression, along with ERRγ. However, GSK5182 strongly inhibited both basal and alcohol-induced hepatic CREBH-N, and CRP protein expression in mice liver (Fig. 5B). Our laboratory reported previously that the diabetic obese condition (*db/db* mice), induces ERRγ gene expression [6]. A significant induction in CREBH-N and CRP protein levels were observed under the same conditions. However, we observed that GSK5182 treatment substantially suppressed both CREBHN, and CRP protein levels in *db/db* mice liver in accordance with previous results (Fig. 5C). Overall, we demonstrated that ERRγ regulates CREBH gene expression *in vitro* and *in vivo*.

## Discussion

ER stress activates the unfolded protein response to generate multiple transcription factors that function in different cellular phenomena including chromatin remodeling [40]–[44]. Our results provide direct evidence for a newly recognized function of ERRγ during transcriptional regulation of CREBH in response to ER stress, as well as in diabetic obese condition [38], [11]. The coactivator PGC1α plays a crucial role in this regulation. Moreover, chromatin remodeling during subsequent transcription events was observed and provided a mechanistic basis for the ERRγ-mediated induction of CREBH under different physiological conditions.

We reported previously that ERRγ directly regulates ATF6α, an ER membrane-bound bZIP transcription factor, in response to ER stress [38]. CREBH is also an ER membrane-bound bZIP transcription factor that belongs to the same CREB3 family as ATF6α [11]. Moreover, we reported an increase in CREBH mRNA level following ERRγ overexpression, although not as much as that of ATF6α [38]. Hence, we investigated the role of ERRγ in the regulation of ER-bound transcription factor CREBH under different physiological conditions including ER stress. We observed significant enhancement of CREBH gene expression by overexpressing ERRγ *in vitro* and *in vivo*. A six-fold increase in CREBH mRNA level was noticed along with a significant increase in the CREBH-N form in response to ERRγ overexpression (Fig. 1). CREBH is regulated by nuclear receptor PPARα, HNF4α, and GR [36]–[37], [15]; hence, our current findings further establish the interconnection between nuclear receptors and ER membrane-bound transcription factors.

According to previous reports, ER stress activates both CREBH and ERRγ gene expression [11], [38]. As we observed CREBH gene induction by ERRγ (Fig. 1), we speculated that ERRγ might be involved in ER stressmediated induction of CREBH. In fact, knockdown of endogenous ERRγ significantly reduced ER stress-induced CREBH mRNA and protein levels (Fig. 2), further supporting the ER stress-ERRγ-CREBH pathway. It has been reported that ERRγ activates target genes by directly binding to the ERRE on the promoter of the target gene [38]. This led us to speculate the presence of ERRE on the CREBH promoter, and we identified a potential site (AGGTCA) on the CREBH promoter for ERRγ binding. The ChIP assay provided further crucial evidence and confirmed ERRγ binding on the CREBH promoter under ER stress. PGC1α is closely associated with ERRγ transcriptional activity [3]. Several lines of evidence have shown that the transcriptional regulation of PDK4 expression by PGC1α is mediated by ERRα or ERRγ [45]. In accordance with these previous reports, occupancy of PGC1α on ERRE of the CREBH promoter was observed during ER stress, providing critical *in vivo* evidence indicating that PGC1α is a coactivator for ERRγ. Gene repression is often associated with decreased histone acetylation [39]. Chromatin remodeling also occurs during coactivator gene transcription [46]. In agreement with previous reports, a significant increase in template-associated histone, H3, and H4 acetylation was noticed (H3 and H4) at the ERRE on CREBH promoter following Tm treatment or ERRγ overexpression, showing the importance of chromatin remodeling during gene transcription (Fig. 3). Overall, our findings reveal a novel molecular mechanism employed by ERRγ linking transactivation of ERRγ [38] to CREBH gene expression. Along with past indications of a probable nuclear receptor/ER membrane-bound transcription factor interconnection, these data raise the possibility (discussed further below) that ERRγ may serve as a key mediator in ER stress-induced CREBH gene expression.

CRP, a known marker for several pathological conditions including ER stress, is regulated by CREBH [11]. Besides being a marker, CRP is an active mediator of endothelial dysfunction, arterial thrombosis, and atherogenesis [24]–[25]. Numerous groups have shown pro-inflammatory and pro-atherogenic effects of CRP *in vitro* [25], [47]–[48]. Transgenic expression of human CRP suppresses endothelial nitric oxide (NO) synthase expression and bioactivity following vascular injury [49]. Several lines of evidence suggest that CRP is a modulator that drives direct biological effects on vascular cells. CRP, at concentrations known to predict adverse vascular events, directly quenches the production of the NO, in part, through a post-transcriptional effect on endothelial NO synthase mRNA stability. Diminished NO bioactivity, in turn, inhibits angiogenesis, an important compensatory mechanism during chronic ischemia [50]. CRP has also been implicated in glucose metabolism. CRP infusion induces an inflammatory response followed by increased norepinephrine and cortisol levels, which result in increased gluconeogenesis [51]. Our initial results (Figs. 1–3) linking ER stress to ERRγ-mediated induction of CREBH, raise the possibility that ERRγ might regulate CREBH target genes by regulating CREBH itself. ERRγ significantly induced CRP gene expression (Fig. 4), which further verifies the ER stress-ERRγ-CREBH pathway. Alcohol consumption induces hepatic ERRγ expression [8]. ERRγ gene expression increases in the liver of *db/db* mice [6]. Moreover, ERRγ along with CREBH are positive regulators of gluconeogenesis [6], [15]. In agreement with these previous reports, alcohol treatment significantly induced both CREBH and CRP gene expression, and this augmentation was substantially compromised upon ERRγ inhibition *in vivo* (Fig. 5B). Regulation of CREBH by ERRγ was further established when GSK5182 treatment significantly reduced CREBH and its target gene, CRP, expression in db/db mice liver tissue (Fig. 5C).

ERRγ, CREBH, and CRP are implicated in numerous biological events. ERRγ regulates hepatic gluconeogenesis [6] and hepatic lipid metabolism [7]. CREBH positively regulates hepatic gluconeogenesis [15]. In addition, CREBH is a master regulator of the lipin 1 gene, a key regulator of lipid metabolism [17]. Both CREBH and ERRγ gene expression is enhanced in a diabetic mouse model to increase blood glucose level [15], [6]. Regulation of both factors is important to regulate gluconeogenesis in response to ER stress, and diabetes. Because the regulation of CRP is also very critical to maintain glucose homeostasis as well as other cellular phenomena including angiogenesis, inhibiting CREBH transactivity and CRP gene expression by inhibiting ERRγ through GSK5182 could be important to relieve disease symptoms. As depicted in Fig. 5D, we hypothesize that ERRγ directly binds to the CREBH promoter along with coactivator PGC1α to regulate CREBH at the transcriptional level in response to ER stress and other physiological conditions, and that CREBH, in turn, regulates its target genes. Overall, our current findings provide insight into a novel chromatin remodeling strategy utilized by ERRγ, and it may be helpful to understand the complex link between nuclear receptors, ER membrane-bound transcription factors, and glucose homeostasis.
