# Correction: Orphan Nuclear Receptor Errγ Induces C-Reactive Protein Gene Expression through Induction of ER-Bound Bzip Transmembrane Transcription Factor CREBH

**DOI:** 10.1371/journal.pone.0125536

**Published:** 2015-05-06

**Authors:** The PLOS ONE Staff

In Fig 3D of the original published article, the panels for the CREBH promoter spanning region -766/-642 for ERR(gamma) ChIP was accidentally also used for the panels PCG1(alpha) and IgG ChIPs for the same region. The authors have replicated the experiment and have provided the original gel images for both the CREBH spanning region -195/-87 and -766/-642 in addition to the revised figure. The revised image does not change the interpretation of the data and the authors apologize for the errors and any inconvenience they may have caused.

**Fig 3:**
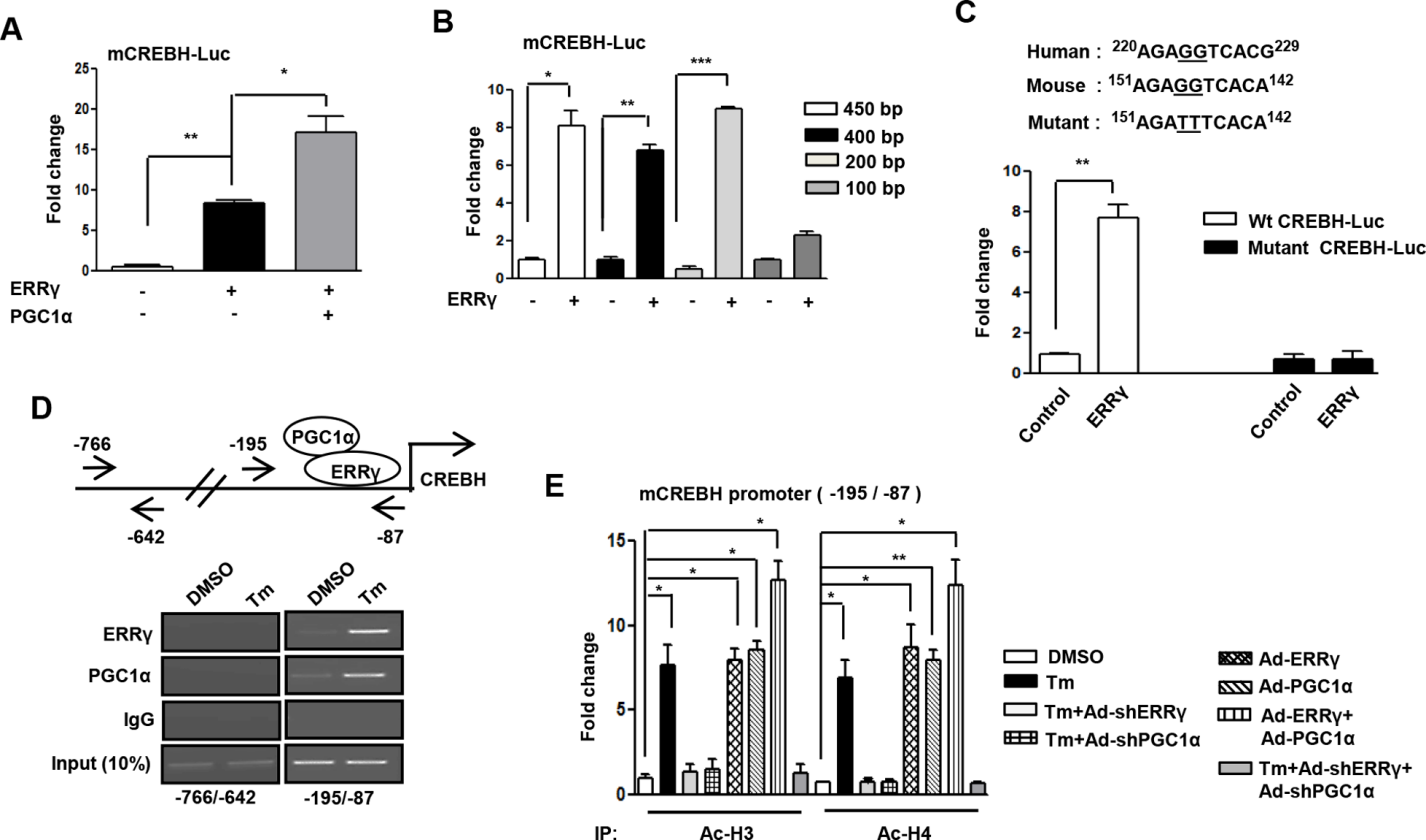
ERRγ regulates activation of CREBH gene promoter. (A), PGC1α-dependent activation of the mouse CREBH promoter by ERRγ. Transient transfection was performed in 293T cells with the indicated plasmid DNAs. (B), Deletion constructs of the CREBH promoter demonstrate the ERRγ binding site in 293T cells. Transient transfection was performed in 293T cells with the indicated plasmid DNAs. (C), ERRE-dependent activation of the CREBH promoter in 293T cells. 293T cells were transfected with the wild-type or ERRE-mutant CREBH promoter along with ERRγ plasmid DNAs. (D), ChIP assay shows the binding of ERRγ and PGC1α to the endogenous CREBH promoter by semiquantitative PCR. AML12 cells were treated with DMSO or Tm (5μg/mL) for 12 hr. After completion of the treatment, chromatin fragments were prepared and immunoprecipitated with ERRγ, PGC1α, or IgG control antibodies. DNA fragments covering –766 to –642 and –195 to –87 elements on the CREBH promoter were PCR amplified. 10% of the soluble chromatin was used as input. (E), ChIP assay for detection of histone acetylation at the ERRγ/PGC1α binding site under the indicated conditions in AML12 cells. Chromatin fragments were prepared and immunoprecipitated with Acetyl-Histone 3 and Acetyl-Histone 4 antibodies. DNA fragments covering –195 to –87 element on the CREBH promoter were qPCR-amplified as described in the ‘Materials and Methods’ section. Data are representative of three independently performed experiments and shown as mean±SD; *P<0.05, **P<0.005, and ***, p<0.0005 using Student’s t-test.

## Supporting Information

S1 File Raw data for Fig 3D (left panel) (TIF)

S2 File Raw data for Fig 3D (right panel) (TIF)

## References

[1] Misra J, Chanda D, Kim D-K, Cho S-R, Koo S-H, Lee C-H, et al (2014) Orphan Nuclear Receptor Errγ Induces C-Reactive Protein Gene Expression through Induction of ER-Bound Bzip Transmembrane Transcription Factor CREBH. PLoS ONE 9(1): e86342 doi: 10.1371/journal.pone.0086342 24466039 10.1371/journal.pone.0086342 PMC3899246

