# Supplementary figures and images for: Correction: Orphan Nuclear Receptor Errγ Induces C-Reactive Protein Gene Expression through Induction of ER-Bound Bzip Transmembrane Transcription Factor CREBH
Source: PLoS One. 2015 May 6;10(5):e0125536. doi: 10.1371/journal.pone.0125536 (PMC4422740; doi:10.1371/journal.pone.0125536)

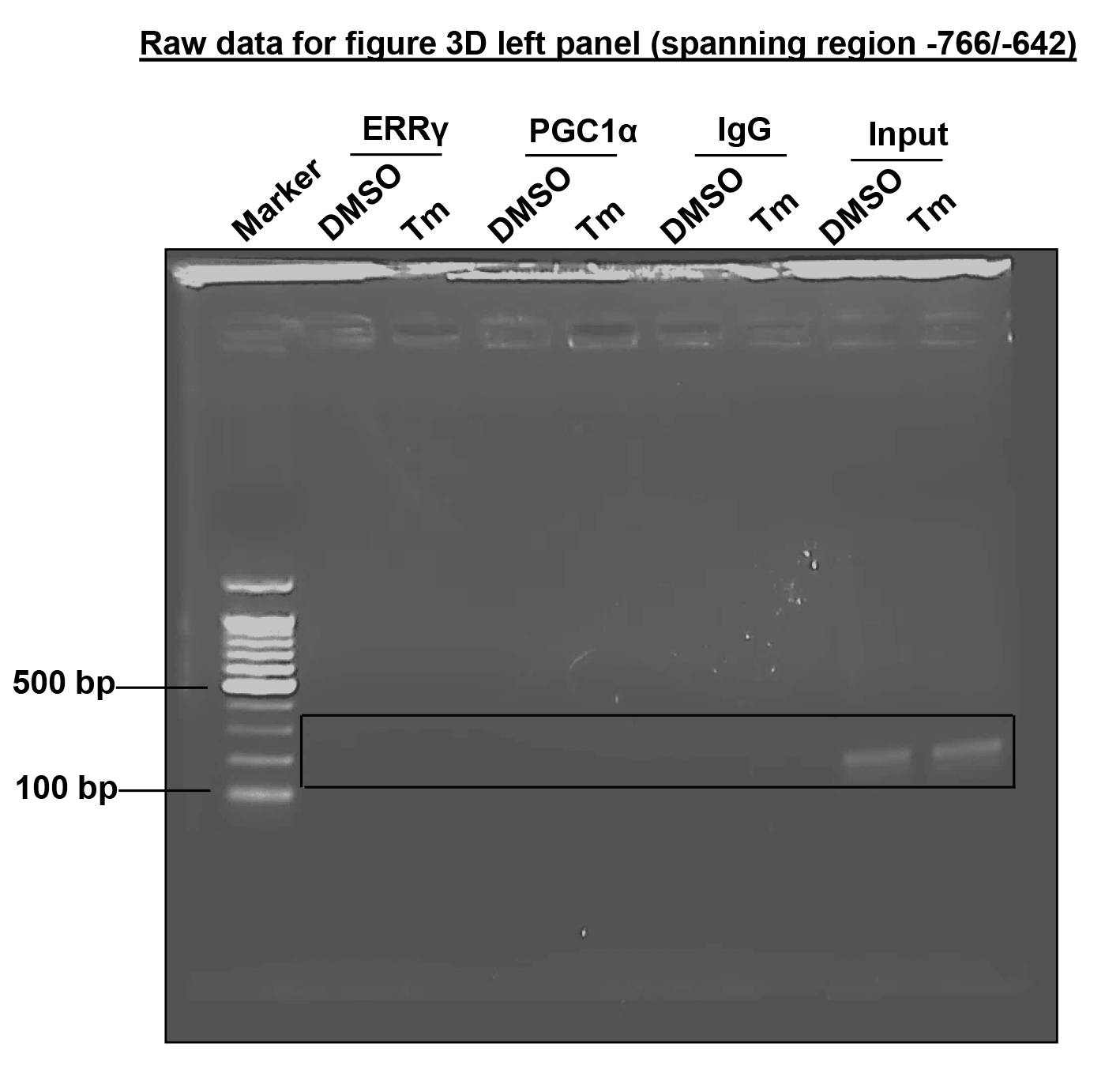

Supplement: S1 File — (TIF) [file pone.0125536.s001.tif]

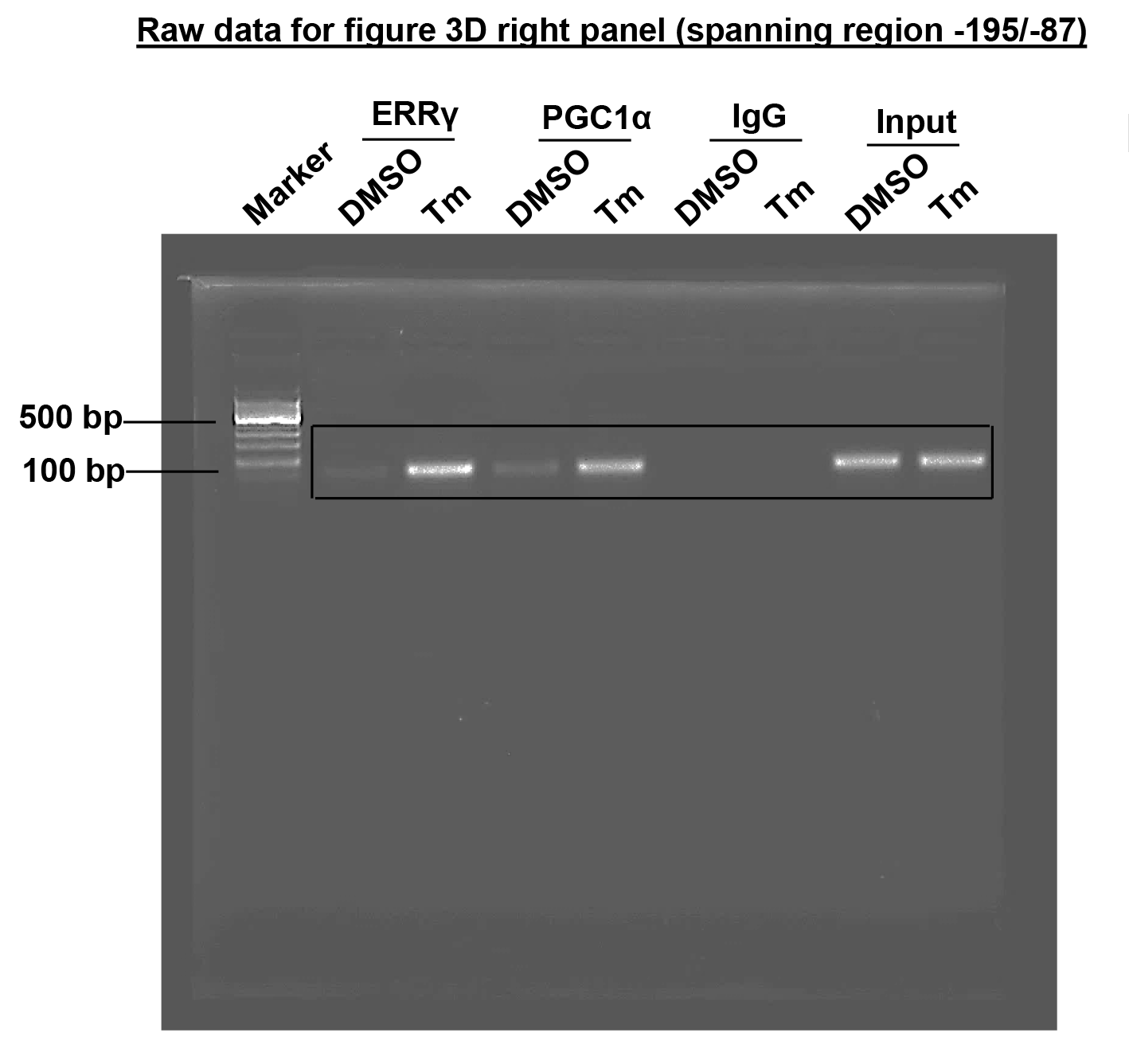

Supplement: S2 File — (TIF) [file pone.0125536.s002.tif]
